# Association of polymorphisms in the heparanase gene (*HPSE*) with hepatocellular carcinoma in Chinese populations

**DOI:** 10.1590/1678-4685-GMB-2014-0338

**Published:** 2017-10-02

**Authors:** Lixia Yu, Xiaoai Zhang, Yun Zhai, Hongxing Zhang, Wei Yue, Xiumei Zhang, Zhifu Wang, Hong Zhou, Gangqiao Zhou, Feng Gong

**Affiliations:** 1 Institute of Transfusion Medicine, Beijing, P. R. China; 2 State Key Laboratory of Proteomics, Beijing Proteome Research Center, Beijing Institute of Radiation Medicine, Beijing, P. R. China

**Keywords:** genetic association, heparanase, hepatocellular carcinoma, *HPSE* gene, PCR-RFLP

## Abstract

Heparanase activity is involved in cancer growth and development in humans and single nucleotide polymorphisms (SNPs) in the heparanase gene (*HPSE*) have been shown to be associated with tumors. In this study, we investigated whether SNPs in *HPSE* were a risk factor for hepatocellular carcinoma (HCC) by undertaking a comprehensive haplotype-tagging, case-control study. For this, six haplotype-tagging SNPs (htSNPs) in *HPSE* were genotyped in 400 HCC patients and 480 controls by polymerase chain reaction-restriction fragment length polymorphism (PCR-RFLP) analysis. A log-additive model revealed significant correlations between the *HPSE* polymorphisms rs12331678 and rs12503843 and the risk of HCC in the overall samples (p = 0.0046 and p = 0.0055). When the analysis was stratified based on hepatitis B virus (HBV) carrier status, significant interactions between rs12331678 and rs12503843 and HBV were observed. Conditional logistic regression analysis for the independent effect of one significant SNP suggested that rs12331678 or rs12503843 contributed an independent effect to the significant association with the risk of HCC, respectively. Our findings suggest that the SNPs rs12331678 and rs12503843 are HCC risk factors, although the potential functional roles of these two SNPs remain to be fully elucidated.

## Introduction

Human hepatocellular carcinoma (HCC), one of the most common tumors in the world, has a high incidence in China, Southeast Asia and sub-Saharan Africa, but a low incidence in the United States and Europe (Ferlay *et al.*, 2010). Malignant growth and metastasis are key features of HCC and are associated with a poor prognosis (Schafer and Sorrell, 1999). During metastasis, tumor cells penetrate the extracellular matrix (ECM) and basement membrane (BM) (Sasisekharan *et al.*, 2002), but adhere poorly to each other; detachment from neighboring cells facilitates entry into blood and lymphatic vessels and dissemination to distant organs.

Heparanase, a mammalian endo-β-D-glycosidase, specifically cleaves the heparan sulphate side chains of heparin sulfate glycosaminoglycans, the most abundant macromolecules in the basement membrane (BM) and ECM (Sasisekharan *et al.*, 2002). Heparanase activity can influence a number of normal and pathological processes, including tissue repair, inflammation, tumor growth, metastasis and angiogenesis (Bishop *et al.*, 2007). In the liver, heparin sulphate-degrading activity is also involved in normal and pathological processes, such as liver development, remodeling and malignant growth (Goldshmidt *et al.*, 2004).

Various studies have examined the clinical significance of heparanase in HCC patients using immunohistochemistry, *in situ* hybridization, RT-PCR and real time-PCR, western blotting and tissue microarrays (TMAs), with the general conclusion being that heparanase is up-regulated in HCC (El-Assal *et al.*, 2001; Xiao *et al.*, 2003; Chen *et al.*, 2004; Liu *et al.*, 2005; Chen *et al.*, 2008). Down-regulating heparanase expression using either antisense oligodeoxynucleotides or RNA interference significantly inhibits the invasiveness, metastasis and angiogenesis of human HCC SMMC7721 cells *in vitro* and *in vivo* (Zhang *et al.*, 2007). Two anti-heparanase antibodies (multiple antigenic peptides MAP1 and MAP2) can effectively inhibit the heparanase activity of HCCLM6 liver cancer cells, thereby influencing their invasive capacity (Yang *et al.*, 2009). Together, these findings indicate that heparanase plays a vital role in HCC metastasis and tumor growth.

The heparanase gene (*HPSE*), located on chromosome 4q21.3, was first cloned in 1999. Several previous reports suggested that single nucleotide polymorphisms (SNPs) in *HPSE* are associated with various types of cancers, including ovarian carcinoma, hematological malignancies, and gastric cancer (Ostrovsky *et al.*, 2007; Ralph *et al.*, 2007; Winter *et al.*, 2008; Yue *et al.*, 2010; Huang *et al.*, 2012; Li *et al.*, 2012). Huang *et al.* (2012) demonstrated that allele loss and reduced *HPSE* expression are closely correlated with tumor progression and poor prognosis in HCC patients. *HPSE* is a tumor suppressor gene based on the fact that tumor suppressor genes usually cause loss of heterozygosity (LOH) in carcinogenesis (Huang *et al.*, 2012). The role of *HPSE* in HCC is currently controversial. In this study, we selected six haplotype-tagging SNPs (htSNPs) distributed throughout the entire gene and investigated whether polymorphisms in *HPSE* were associated with the risk of HCC in a Chinese population.

## Materials and Methods

### Subjects

This case-control study consisted of 400 incident patients with HCC and 480 healthy controls. All subjects were Chinese recruited in Fusui territory and surrounding regions in Guangxi province, a high-risk region for HCC in southern China. The diagnosis of cases, the inclusion and exclusion criteria, the definition of hepatitis B virus (HBV) carriers, smokers and drinkers were as described previously (Zhai *et al.*, 2006, 2007). Thirty-four of the original 434 cases were excluded because genomic DNA was depleted in the original study (Zhang *et al.*, 2010); the remaining 400 cases were included in the study (Table 1). There were no significant differences between the initial 434 and final 400 patients in terms of age and sex distributions (mean ages: 49.1 and 49.3 years, respectively; male/female ratio: 6.6 and 6.5, respectively). Informed consent was obtained from each subject at recruitment and personal information on demographic factors, medical history, tobacco and alcohol use and family history of HCC was collected via a structured questionnaire. This study was approved by the Medical Ethical Committee of the Chinese National Human Genome Center.

**Table 1:** Selected clinical and demographic characteristics of the subjects in the case and control population.

Variable		Patients with HCC (n = 400)	Controls (n = 480)	p
Age, years				
	Mean (SD)	49.3 (12.0)	48.6 (10.0)	0.342†
	≤ 49, n (%)	201 (50.3)	264 (55.0)	0.16‡
	> 49, n (%)	199 (49.7)	216 (45.0)	
Sex, n (%)				
	Male	347 (86.7)	384 (80.0)	0.008‡
	Female	53 (13.3)	96 (20.0)	
Nationality, n (%)				
	Han	256 (64.0)	359 (74.8)	0.001‡
	non-Han^a^	144 (36.0)	121 (25.2)	
Smoking status, n (%)				
	Smoker	162 (40.5)	152 (31.7)	0.006‡
	Nonsmoker	238 (59.5)	328 (68.3)	
Smoking level, pack-years				
	Mean (SD)	22.1 (14.1)	19.1 (15.9)	0.08†
	≤ 23, n (%)	102 (63.0)	100 (65.8)	0.60‡
	> 23, n (%)	60 (37.0)	52 (34.2)	
Alcohol use, n (%)				
	Drinker	116 (29.0)	145 (30.2)	0.696‡
	Nondrinker	284 (71.0)	335 (69.8)	
HBV carriers, n (%)				
Yes		303 (75.7)	186 (38.8)	< .001‡
No		97 (24.3)	294 (61.2)	
First-degree family history of HCC, n (%)				
	Positive	74 (18.5)	26 (5.4)	< .001‡
	Negative	326 (81.5)	454 (94.6)	

a Non-Han individuals included Zhuang (n = 139), Jing (n = 1) and Yao (n = 4) nationalities. In the controls, all of the non-Han individuals were of the Zhuang nationality (n = 121). HBV – hepatitis B virus, HCC – hepatocellular carcinoma, SD – standard deviation.

† *P* value for two-tailed *t-*test.

‡ *P* value for two-tailed χ^2^ test.

### Selection of haplotype-tagging SNPs (htSNPs)

**Figure 1:**
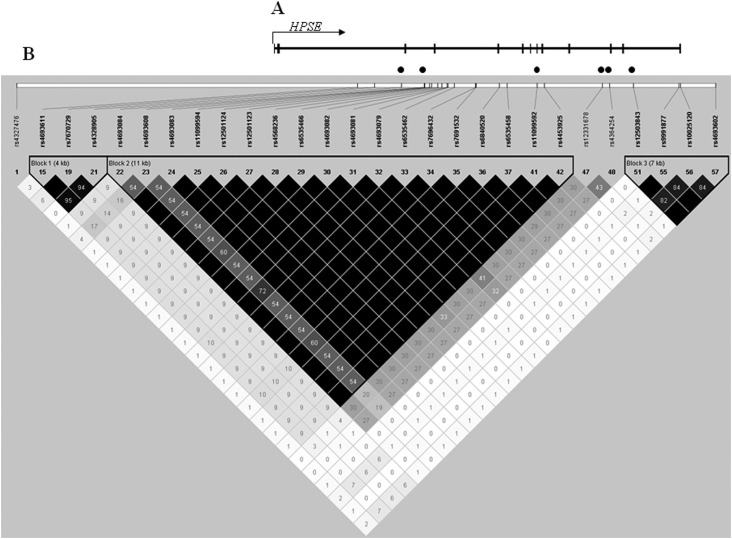
SNPs in the region of *HPSE* located on chromosome 4q21.3. (A) *HPSE* gene structure. Vertical bars represent the 13 exons (5’ to 3’). The arrow shows the transcription start site and direction of gene transcription. Solid black points indicate the location of htSNPs in the gene. (B) Diagram of the block structure of *HPSE* on chromosome 4q21.3 generated with Haploview software. The value within each diamond represents the pairwise correlation between tagging SNPs (measured as D’) defined by the upper left and the upper right sides of the diamond. The strength of the linkage disequilibrium (LD) is shown in increasing shades of gray. The diamonds without a number correspond to D’ = 1. Shading represents the magnitude and significance of pairwise LD. Selected htSNPs in three major blocks of *HPSE* are shown.

The htSNPs and candidate SNPs were selected to allow complete *HPSE* gene coverage and to replicate previously reported associations. The htSNPs were selected from genotyped SNPs in the Han Chinese population (HCB) of the HapMap project (Phase II database) by using a haplotye-tagging SNP approach (Couzin, 2006), with a minor allele frequency ≥ 5%. The selected htSNPs had an estimated correlation coefficient (r^2^) of > 0.8. Candidate SNP rs11099592, as a non-synonymous coding SNP, was selected based on previously reported associations with hematological malignancies (Ostrovsky *et al.*, 2007). Six htSNPs across the 4.9-kb region spanning *HPSE* loci on chromosome 4q21.3 (84430497-84480330; NCBI Build 37, hg18), from 5 kb upstream to 5 kb downstream of *HPSE*, were selected for genotyping. These tagging polymorphisms were rs4328905 (intron2), rs4693608 (intron3), rs11099592 (exon8), rs4364254 (intron10), rs12331678 (intron10) and rs12503843 (intron12), which were distributed throughout the full length of the gene. rs12331678 and rs4364254 could not be included in the blocks. The linkage disequilibrium (LD) plot for the full *HPSE* gene is shown in Figure 1.

### Genotyping

Genomic DNA from peripheral blood leukocytes was extracted from 5 mL of whole blood by using standard phenol/chloroform protocols. DNA samples were diluted to 10 ng/μL and distributed onto 96-well plates, each of which contained 94 samples and two wells of no DNA-control water.

The six htSNPs in *HPSE* were genotyped in our case-control populations using a PCR-RFLP (polymerase chain reaction-restriction fragment length polymorphism) assay. The PCR fragments covering the relevant htSNPs were amplified via PCR from genomic DNA and the amplicons then digested with an appropriate restriction enzyme that specifically cleaved one allele. The digestion products were subjected to gel electrophoresis and visualized under UV light via Goldview staining. The PCR primers used in the PCR-RFLP assays and the appropriate restriction enzymes are shown in Table 2. Genotyping was done by staff members who were blinded to the subjects’ case or control status. The accuracy of the genotyping data for each polymorphism obtained via the PCR-RFLP analyses was validated by the direct sequencing of a 15% masked random sample of cases and controls, and all of the results showed 100% concordance.

**Table 2:** Primers and restriction enzymes used in *HPSE* polymorphism genotyping by PCR-RFLP.

Polymorphisms	Modification	Primer sequence	Amplicon size (bp)	Restriction enzyme	Digest size (bp)
rs4328905	T- > C	5’- CAGGAGGCAGAAGCAGAACT-3’ (forward)	250	*Taa*I	T/T: 250
		5’- TGTTAGGAAGACAAGCAAGAAAGT-3’ (reverse)			C/C: 221, 29
					T/C: 250, 221, 29
rs4693608	A- > G	5’-GTGCCTGACCAGGGTGGAT-3’ (forward)	204	*Hinc*II	A/A: 204
		5’- GTGCAGTTCTCTTAAGGTTCACTT −3’ (reverse)			G/G: 118, 86
					G/A: 204, 118, 86
rs11099592	T- > C	5’-GTTACTTGCATAGAATGGACTCCTC-3’ (forward)	250	*Bst*NI	T/T: 250
		5’-TGTCCAATACATCAGGGTTTAGAAAA-3’ (reverse)			C/C: 216, 34
					T/C 250, 216, 34
rs12331678	A- > C	5’-CTATAGTATTTCCTACATTATAGAGTTTGGTA-3’ (forward)	240	*Rsa*I	A/A: 240
		5’- TGGATTAGGCAATGGTCATCA-3’ (reverse)			C/C: 209, 31
					A/C: 240, 209, 31
rs4364254	T- > C	5’- TACCCACTTCAGCTTCCCAAA-3’ (forward)	226	*Eco*RV	T/T: 226
		5’-GTCAAGAATGATCAGAGTTTAAGTATTCTTGGATAT-3’ (reverse)			C/C: 192, 34
					T/C: 226, 192, 34
rs12503843	T- > C	5’-AAAGCAAAAGGATGTGAACACAAA-3’ (forward)	261	*Mnl*I	T/T: 261
		5’-CTTACTCTAACCAATAAAAATTAATGCTATAGA-3’ (reverse)			C/C: 237, 24
					T/C: 261, 237, 24

### Statistical analysis

Genotype and allele frequencies for *HPSE* polymorphisms were determined by gene counting. The degree of fit to Hardy-Weinberg equilibrium was tested using the χ^2^ test. Multiple logistic regression analyses were used to evaluate the associations between the polymorphisms and the risk of HCC and were adjusted to account for confounding factors (including age, sex, smoking and drinking status, and level of smoking); the p values, odds ratios (ORs) and 95% confidence intervals (95% CIs) were then calculated. Potential modification of the effect of the polymorphisms on the risk of HCC by the possible confounding factors was assessed by adding interaction terms to the logistic model and by stratification analyses of subgroups of subjects determined by these factors. All statistical analyses were done with SPSS software (version 10.0, SPSS Inc.). SNP spectral decomposition was used to calculate the M_eff_ value and thus correct for multiple testing (an LD-based method, available at http://gump.qimr.edu.au/general/daleN/SNPSpD/). This correction strategy accounted for the LD between polymorphic sites. Since failure to account for the non-independence of SNPs would make the Bonferroni correction over-conservative, a value of p < 0.01 (0.05/5) was considered to be statistically significant (Nyholt, 2004).

## Results

Table 1 compares the characteristics of the cases and controls. The two groups were comparable with regard to age, drinking status and pack-years of smoking (p > 0.05). However, compared with controls, there were more men (p = 0.008), smokers (p = 0.006), HBV carriers (p = 0.001) and patients with a history of HCC among their first-degree biological relatives (p = 0.001) in the cases group.

The genotype and allele distributions of the six htSNPs between patients with HCC and the controls are summarized in Table 3. The genotype distributions for the six htSNPs were in Hardy-Weinberg equilibrium in each group (p = 0.05). Significant correlations were found between the SNPs rs12331678 and rs12503843 and the risk of HCC in the overall samples (adjusted OR, 1.69; 95% CI, 1.17-2.43; p = 0.0046; adjusted OR, 1.52; 95% CI, 1.13-2.05; p = 0.0055) in the log-additive model. The level of significance was maintained after correcting for multiple testing (SNPSpD). When the analyses were stratified by HBV status, the SNP rs12503843 was significantly associated with the susceptibility to HCC in HBV carriers (p = 0.030) in the log-additive model. There were associations between rs12331678 and the risk of HCC in non-HBV carriers (adjusted OR, 2.19; 95% CI, 1.25-3.85; p = 0.0074) in the log-additive model, but no associations were found in HBV carriers. An elevated frequency of the A allele for rs12331678 was observed in patients with HCC. The A allele frequency was significantly higher in patients than in controls (0.11 *vs.* 0.074, p = 0.006). The rs12503843-T allele may be also a risk factor for HCC (0.16 *vs.* 0.11, p = 0.003). Subjects bearing the rs12331678-A or rs12503843-T allele had an increased risk of HCC (Table 4).

**Table 3:** Results of the association test for the *HPSE* polymorphism genotype between HCC patients and controls.*

SNP	Case, N (%)/Control, N (%)	Codominant		Dominant		Recessive		Log-additive	
		OR (95% CI)	p	OR (95% CI)	p	OR (95% CI)	p	OR (95% CI)	p
rs4328905									
TT	136 (36.1)/179 (37.4)		0.70	1.06 (0.77-1.44)	0.73	1.19 (0.80-1.76)	0.40	1.07 (0.88 to 1.30)	0.49
TC	170 (45.1)/216 (45.2)	1.01 (0.72 to 1.41)							
CC	71 (18.8)/83 (17.4)	1.19 (0.77 to 1.84)							
C	312 (41.4)/382 (40.0)								
rs4693608									
AA	246 (64.4)/329 (68.7)		0.4	1.22 (0.89 to 1.67)	0.22	0.92 (0.42 to 2.02)	0.83	1.16 (0.90 to 1.49)	0.33
GA	122 (31.9)/134 (28.0)	1.25 (0.90 to 1.74)							
GG	14 (3.7)/16 (3.3)	0.98 (0.44 to 2.19)							
G	150 (19.6)/166 (17.3)								
rs11099592									
CC	316 (80.4)/386 (81.8)		0.74	1.16 (0.79 to 1.70)	0.44	1.13 (0.34 to 3.82)	0.84	1.12 (0.82 to 1.52)	0.46
TC	71 (18.1)/79 (16.7)	1.16 (0.78 to 1.72)							
TT	6 (1.5)/7 (1.5)	1.17 (0.35 to 3.93)							
T	83 (10.6)/93 (9.8)								
rs12331678									
CC	306 (79.1)/410 (89.6)		**0.015**	1.69 (1.14 to 2.49)	**0.0086**	4.19 (0.74 to 23.59)	0.083	1.69 (1.17 to 2.43)	**0.0046**
CA	75 (19.4)/67 (14.0)	1.60 (1.07 to 2.39)							
AA	6 (1.5)/2 (0.4)	4.55 (0.81 to 25.67)							
A	87 (11.2)/71 (7.4)								
rs4364254									
TT	173 (44.9)/223 (46.5)		0.65	1.11 (0.83 to 1.50)	0.47	0.92 (0.57 to 1.49)	0.73	1.03 (0.84 to 1.26)	0.7
TC	173(44.9)/204 (42.5)	1.15 (0.84 to 1.57)							
CC	39 (10.1)/53 (11.0)	0.99 (0.60 to 1.63)							
C	251 (32.6)/310 (32.3)								
rs12503843									
CC	273 (71.3)/381 (79.4)		**0.021**	1.59 (1.13 to 2.25)	**0.0081**	2.07 (0.82 to 5.25)	0.12	1.52 (1.13 to 2.05)	**0.0055**
CT	96 (25.1)/90 (18.8)	1.52 (1.06 to 2.19)							
TT	14 (3.6)/9 (1.9)	2.29 (0.90 to 5.81)							
T	124 (16.2)/108 (11.3)								

* The number of genotyped samples varied because of genotyping failure for some individuals. CI – confidence interval, OR – odds ratio. ORs and 95% CIs were calculated by logistic regression using an additive model and were adjusted for age, sex, status of smoking and drinking, smoking levels and HBV carrier status. The HBV carriers were positive for both HBsAg and HBcAb for at least 12 months. Significant deviations (p < 0.01) are shown in bold after correction for multiple comparisons.

**Table 4:** Stratification of rs12331678 and rs12503843 based on HBV carrier status in HCC patients and controls.

Confounding factor	SNP	Case, N (%)/Control, N (%)	Codominant		Dominant		Recessive		Log-additive	
			OR (95% CI)	p	OR (95% CI)	p	OR (95% CI)	p	OR (95% CI)	p
HBV carriers	rs12331678									
	CC	235 (79.9)/157 (84.4)		0.37	1.39 (0.84-2.30)	0.19	2.27 (0.24-21.36)	0.44	1.39 (0.87-2.21)	0.16
	CA	55 (18.7)/28 (15.0)	1.36 (0.81-2.26)							
	AA	4 (1.4)/1 (0.5)	2.40 (0.25-22.69)							
	rs12503843									
	CC	207 (71.1)/147 (79.0)		0.095	1.60 (1.02-2.49)	**0.037**	2.00 (0.61-6.49)	0.23	1.51 (1.03-2.21)	**0.030**
	CT	73 (25.1)/35 (18.8)	1.53 (0.96-2.43)							
	TT	11 (3.8)/4 (2.2)	2.20 (0.67-7.19)							
Non-HBV carriers	rs12331678									
	CC	71 (76.3)/253 (86.4)		**0.023**	2.19 (1.19-4.04)	**0.014**	7.95 (0.69-91.56)	**0.086**	2.19 (1.25-3.85)	**0.0074**
	CA	20 (21.5)/39 (13.3)	2.02 (1.08-3.79)							
	AA	2 (2.2)/1 (0.3)	8.99 (0.78-103.63)							
	rs12503843									
	CC	66 (71.7)/234 (79.6)		0.32	1.50 (0.86-2.63)	0.16	2.10 (0.46-9.59)	0.35	1.46 (0.90-2.38)	0.13
	CT	23 (25.0)/55 (18.7)	1.44 (0.80-2.58)							
	TT	3 (3.3)/5 (1.7)	2.29 (0.50-10.50)							

CI – confidence interval, HBV – hepatitis B virus, OR – odds ratio. ORs and 95% CIs were calculated by logistic regression using an additive model and were adjusted for age, sex, status of smoking and drinking and smoking levels. Significant deviations (p < 0.05) are shown in bold after correction for multiple comparisons.

In a multiple logistic regression analysis, rs12331678 and rs12503843 were significantly associated with HCC when adjusted for the effect of rs12503843 and rs12331678 (residual p = 0.011 and 0.0062 respectively; Table 5).

**Table 5:** Independent effects of rs12331678 and rs12503843 in the overall populations.

Polymorphisms	Cases, n (%)	Controls, n (%)	OR (95% CI)^a^	p*a*	OR (95% CI)^b^	p*b*
rs12331678						
C/C	300 (79.4)	410 (85.6)	1.62 (1.16-2.26)	0.0042	1.55 (1.10-2.17)	**0.011**
A/C	72 (19.1)	67 (14)				
A/A	6 (1.6)	2 (0.4)				
rs12503843						
C/C	271 (71.7)	381 (79.5)	1.53 (1.16-2.01)	0.0023	1.47 (1.12-1.95)	**0.0062**
T/C	94 (24.9)	89 (18.6)				
T/T	13 (3.4)	9 (1.9)				

CI – confidence interval, OR – odds ratio. The p values, ORs and 95% CIs were calculated by logistic regression using an additive model and were adjusted for age, sex, status of smoking and drinking, smoking levels and HBV carrier status.

a,b Values before (^a^) and after (^b^) adjustment for rs12503843 or rs12331678. Significant deviations (p < 0.05) are shown in bold.

## Discussion

Our study comprehensively assessed common variations in the *HPSE* region that were excluded in our GWAS study (Zhang *et al.*, 2010). In the present study, we found that two polymorphisms in the *HPSE* gene, rs12331678 in intron 10 and rs12503854 in intron 12, were significantly associated with susceptibility to HCC in a Chinese population. The associations between SNPs and risk of HCC were further examined with stratification based on HBV carrier status. There was a significant interaction between rs12503843 and HBV carrier status, suggesting that this status modified the susceptibility to HCC related to the rs12503843 genotype. There was also a significant association between rs12331678 and risk of HCC in non-HBV carriers. Conditional logistic regression analysis for the independent effect of one significant SNP adjusted by the other SNP suggested that rs12331678 or rs12503843 contributed an independent effect to the significant association with the risk of HCC, respectively. These findings confirmed the initial hypothesis that the *HPSE* gene may play an important role in the pathogenesis of HCC.

The current view is that some functionally important non-coding variants may qualitatively or quantitatively alter gene expression (Wang *et al.*, 2006). The polymorphism rs12331678 in intron 10, located at a block-block boundary, showed weak linkage disequilibrium with the other variants in blocks 2 and 3. This result suggests the polymorphism rs12331678 may be a causative variant. Indeed, the type of allele (C or A) predicts the changes in regulatory function for loci in terms of the transcription of *HPSE*. Bioinformatics prediction of the change in transcriptional factors was done using Alibaba2.1 software. Risk allele rs12331678 [A] can bind the factor SP1 (specificity protein 1), but the C allele does not. SP1, a nuclear transcription factor, plays an extremely important role in the growth and metastasis of many tumors, including HCC, by regulating growth-related signal transduction, angiogenesis-related pathways and other factors. Thus, the alleles of rs12331678 may be involved in the regulation of *HPSE* expression, although the potential functional roles of this SNP remain to be fully elucidated.

The potential mechanisms involved in the association between rs12503843 and the risk of HCC may include the ability of this SNP to exert a functional role and to act as a marker in tight LD with other functional SNPs in the *HPSE* 3’UTR. Ostrovsky *et al.* (2007) have shown that SNP rs4693602, which maps to a distal part of the 3’-UTR of the *HPSE* gene, was associated with multiple myeloma (MM) and may modify *HSPE* expression. The intronic polymorphism rs12503843 is in tight LD with rs4693602 (see Figure 1) and hence the intron 12 SNP might act as a genetic marker, possibly because it too is in tight LD with another SNP downstream in the *HPSE* 3’-UTR region.

SNP rs11099592 is an A-G replacement located in the coding region of *HPSE* (exon 8) that results in the substitution of arginine for lysine at position 307. In contrast to the findings of Ostrovsky *et al.* (2007), no association was observed here between the SNP rs11099592 and the risk of HCC in the log-additive model (adjusted OR, 1.12; 95% CI, 0.82-1.52; p = 0.46), a finding consistent with the results of Winter *et al.* (2008). However, arginine (rs11099592-A) and lysine (rs11099592-G) are basic amino acids, and this type of modification might not cause an obvious functional distortion of activated heparanase; this situation may have contributed to the lack of an association between rs11099592 and HCC. Another possible explanation is that different diseases and population-specific variations may involve different genetic mechanisms of susceptibility.

Previous work suggested that rs4693608, as a functional polymorphism, was strongly correlated with the risk of acute graft-versus-host disease (GVHD) (Ostrovsky *et al.*, 2013). However, our negative results could be explained by differences in the genetic effect among ethnic groups, *e.g.*, population differences in the LD pattern or allele frequencies of *HPSE*. For example, there may be a small, population-specific effect of *HPSE* rs4693608 on the development of HCC. Indeed, the allelic and genotypic frequencies of *HPSE* rs4693608 vary with ethnicity. For instance, the frequencies of the rs4693608 G allele and GG genotype were 0.17 and 0.03, respectively, in our study with 480 controls compared to 0.46 and 0.17 in Israeli (Ostrovsky *et al.*, 2014). Ethnic variation in the distribution of the *HPSE* genotype warrants additional comparative studies in other populations of different ancestry, such as Caucasians and Israelis, to confirm our results.

rs4328905 located in intron 2 of *HPSE* showed no association with the risk of HCC in our study. Likewise, Yue *et al.* (2010) and Li *et al.* (2012) reported no association between rs4328905 and the risk of gastric cancer. Together, these findings indicate that rs4328905 is not correlated with the incidence of cancers such as HCC and gastric cancer. Similarly, the association between rs4364254 and the risk of HCC risk was not significant in the present study. Ostrovsky *et al.* (2010) provided the first evidence of a correlation between rs4364254 and the risk of GVHD in a recessive model. In addition, individuals with rs4364254-TT possessed relatively high mRNA levels (p = 0.0029) (Ostrovsky *et al.*, 2009) and there was a significant correlation with poor survival (p = 0.013) (Li *et al.*, 2012). These apparent discrepancies may be related to differences among diseases and the relatively low number of patients included in the studies.

Our data revealed two putative *HPSE* SNPs associated with a risk of HCC. However, several limitations of this study should be noted First, as a hospital-based study, our HCC cases were selected from hospitals whereas the controls were selected from a community population so that inherent selection bias cannot be completely excluded. With further adjustment and stratification in the data analyses, potential confounding factors could have been minimized. Second, although the highly significant association between *HPSE* and the risk of HCC was derived from a biologically-based *a priori* hypothesis, our initial findings need to be verified independently in other populations with high incidences of HCC, such as other southern Chinese, as well as Singaporeans and Taiwanese. Lastly, the role of intronic SNPs should be taken into account.

In conclusion, we evaluated the relationship between *HPSE* gene polymorphisms and susceptibility to HCC in a southern Chinese population. Two intronic SNPs were found to be correlated with a risk of HCC, but the molecular mechanisms linking these noncoding variants with HCC are still unclear. Direct connection between HCC-associated variants and heparanase expression or function should be explored in future studies.
